# The impact of cavaletti height on dogs’ walking speed and its implications for ground reaction forces

**DOI:** 10.3389/fvets.2024.1419206

**Published:** 2024-07-23

**Authors:** Cara A. Blake, Andrea L. Looney, Tracie D. Merrill

**Affiliations:** ^1^Central Hospital for Veterinary Medicine, Guilford, CT, United States; ^2^Central Hospital for Veterinary Medicine, North Haven, CT, United States; ^3^Reese Chiropractic, Stillwater, OK, United States

**Keywords:** cavaletti pole, rehabilitative therapy, pressure-sensitive walkway, kinetics, dog

## Abstract

**Objective:**

The objective of this study was to investigate the effects of cavaletti pole height on temporospatial (TPS) and ground reaction force (GRF) variables as compared to a walking gait in healthy dogs.

**Animals:**

A total of 25 client-owned dogs were included in this study.

**Procedures:**

This study used client-owned dogs to explore the effects of cavaletti pole height on TPS and GRF variables. Dogs were first walked over a validated pressure-sensitive walkway (PSW) and then walked over the PSW over which six cavaletti poles were set. Cavaletti pole height was initially set at 2 inches and then increased incrementally to 4 inches, 6 inches, and 8 inches. TPS and GRF variables were obtained for all dogs walking across a PSW without cavaletti poles and at each cavaletti height. TPS variables were then compared to those obtained at a normal walking gait.

**Results:**

Increasing cavaletti height resulted in significant decreases in walking gait velocity and the number of gait cycles per minute. Conversely, significant increases in gait cycle duration (duration of one complete cycle of gait, which includes the time from the initial contact of one paw to the subsequent contact of the same paw) and gait time (duration to walk the total distance on the PSW) were noted. Increases in stance time, normalized maximum force, and normalized vertical impulse were observed.

**Conclusion and clinical relevance:**

Cavaletti height does influence TPS variables in healthy dogs at a walking gait. The effects were most notable with regard to velocity. Due to the lack of consistent velocity for all cavaletti heights, no conclusions can be drawn regarding the effect of cavaletti height on ground reaction forces. Further investigation is needed to elucidate whether it is the velocity, cavaletti height, or combination of both that impacts ground reaction force variables. When selecting cavaletti pole heights for a therapeutic exercise program, an increase in cavaletti height results in a slower walking gait.

## 1 Introduction

Objective gait analysis has gained significant attention in veterinary medicine due to its relevance to the understanding of locomotion and identifying gait abnormalities in companion animals. Force plates and pressure-sensitive walkways have been used to evaluate the kinetics of the canine gait in both research and clinical settings (1–17). Force-plate (FP) systems provide ground reaction force (GRF) information for one limb or footfall. Pressure-sensitive walkways (PSW) measure temporospatial (TPS) and ground reaction forces (GRF) information about all four limbs and multiple gait cycles (18). PSWs have been used to characterize the TPS and GRFs in different populations of dogs under various conditions (19–30).

Rehabilitative therapy is an evolving discipline within the field of veterinary medicine. There has been tremendous growth in this field, and a previous study reported that approximately 70% of veterinarians refer patients for rehabilitation (31). Therapeutic exercises are an important component of veterinary rehabilitation programs. Changes in weight bearing status are used to modify and progress therapeutic exercises in veterinary patients. Cavaletti poles are commonly included as part of a therapeutic exercise program to improve joint range of motion, balance, coordination, proprioception, and weight bearing. Cavaletti poles are typically set at a low height initially, and as the patient progresses, the pole height is increased. Additionally, cavaletti poles of varied heights, spacing, and layouts can be utilized to increase the difficulty of the exercise (32–40).

Walking over obstacles has been researched in human subjects. These human studies have shown that negotiating obstacles during locomotion is a multifaceted process that demands coordinated efforts from various physiological systems (41). Upon approaching an obstacle, its dimensions and surface properties are evaluated to formulate an ideal strategy for crossing (42). Limbs are raised, and joints are flexed and extended to clear the object. During these moments, equilibrium is sustained through the activation of core muscles and subtle adjustments in posture and limb alignment. Depending on the obstacle’s size and characteristics, adaptations in gait patterns or step lengths may be warranted to ensure adequate clearance (43–48). In quadrupeds, such adjustments may entail varying degrees of articulation in the thoracic and pelvic limbs (49–51).

Bipedal and quadrupedal obstacle walking requires the negotiation of barriers but diverges in limb usage, stability, biomechanics, and energy expenditure. Bipedal locomotion, relying on two limbs, entails heightened instability and places greater demands on the musculoskeletal system (41, 42). In contrast, quadrupedal locomotion, leveraging four limbs, offers enhanced stability and energy efficiency (49–51).

When a bipedal animal confronts a vertical obstacle, the leading limb starts the movement, lifting and clearing the barrier, with the trailing limb providing stability and reinforcement. This synchronized interplay between the leading and trailing limbs facilitates agile obstacle negotiation while maintaining equilibrium (52). Conversely, in quadrupedal locomotion, a dynamic interplay occurs among the leading forelimb, trailing forelimb, leading hindlimb, and trailing hindlimb, each fulfilling specialized roles to ensure smooth traversal over vertical obstacles (49–51).

Studies have evaluated the kinematics, kinetics, and muscle activation during walking, trotting, and jumping over obstacles in dogs and horses (53–60). The effect of fence height, increasing hurdle heights, and differing distances between obstacles on jump kinematics has been reported in dogs (55–57). A study evaluating hindlimb kinematics in dogs with hip osteoarthritis when walked over carpus-height obstacles revealed changes in stifle and tarsal joint range of motion but no changes in hip joint kinematics (58). In studies investigating surface electromyography in dogs walking over obstacles, increased muscle activity of the vastus lateralis and gluteus medius was noted (59, 60).

Despite the growth in rehabilitative therapy for veterinary patients, there is still a lack of information regarding the specific exercises used in therapeutic exercise programs. Limited information is available on the gait kinetics of canines when walking over obstacles (59–62). A recent study investigated the effects of walking over one or two obstacles on ground reaction forces and the center of pressure (COP) within the paws of healthy dogs. The results demonstrated slower walking speeds, increased vertical impulse during the stance phase of the pelvic limbs, and changes in the COP when compared to walking without obstacles (63). To the authors’ knowledge, there are no previously reported data published in the literature reporting information with regard to dogs walking over multiple sequential obstacles, such as cavaletti poles. The paucity of data leaves the veterinary rehabilitation practitioner to base parameters for cavaletti pole exercises on clinical experience and extrapolation from studies on other species (human and rat). Therefore, the goal of this study was to examine the impact of walking over multiple obstacles (cavaletti poles) set at increasing heights on TPS and GRF parameters in healthy dogs during a walking gait. We hypothesized that there would be differences in both TPS and GRF variables with increasing cavaletti pole height when compared to a walking gait.

## 2 Materials and methods

The study was approved by the Institutional Animal Care and Use Committee at Oklahoma State University. Client- and staff-owned dogs were recruited to participate in this study, and written owner consent was obtained prior to enrollment.

A complete physical, neurologic, and orthopedic exam was performed on all dogs by a board-certified veterinary surgeon (CAB). The breed, age, sex, weight, and body condition score (BCS, 1–9) were recorded. The height at the withers was measured using a commercial measuring stick^1^ and recorded. Dogs were excluded from the study if they had evidence of orthopedic or neurologic disease or other systemic diseases that would adversely affect locomotion, were not amenable to leash walking, were not amenable to walking over cavaletti poles, were not amenable to walking over pressure-sensitive walkway, and/or had a measured wither height of <50 cm or > 65 cm.

A PSW system^2^ was used to obtain temporospatial gait and GRF measurements. The PSW was calibrated as per the manufacturer’s instructions using a phantom of known weight. Data were transmitted to a dedicated computer using Tekscan software (Strideway™ version 7.7) and subsequently exported to Microsoft Excel.

**Figure 1:**
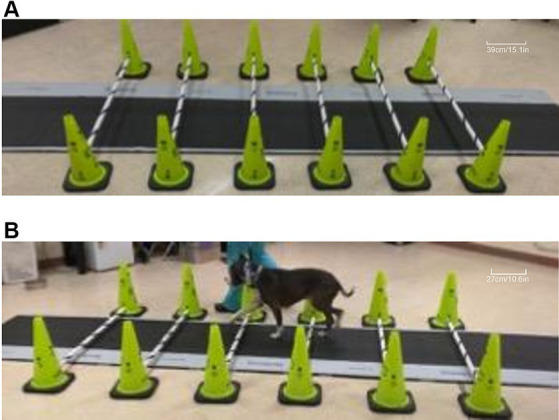
Images of cavaletti pole and pressure-sensitive walkway (PSW) setup. A representative image depicting six cavaletti poles set at the 6-inch (15.2 cm) height setup over the centrally placed PSW **(A)**. A dog with a measured withers height of 54 cm is walked on the left-hand side of the handler over calvaletti poles spaced 54 cm apart, set at the 2” (5.1 cm) cavaletti height that were placed over the PSW **(B)**.

Prior to data acquisition, each dog was allowed to adapt to the room where the gait analysis was performed. Once comfortable, each dog was leash-walked around the room, over the pressure-sensitive walkway (PSW), over cavaletti poles, and over cavaletti poles that were set up over the PSW (Figure 1A). Dogs were walked on leash by the same handler (TDM), on the left-hand side of the handler. Each dog was walked at their preferred velocity over the PSW to obtain a baseline gait evaluation at a walking gait. The dogs were then walked over the PSW, at their preferred velocity, over which six cavaletti poles were set (Figure 1B). Cavaletti poles^3^ were initially set at a height of 2 inches (5.1 cm) and heights were incrementally increased to 4 inches (10.2 cm), 6 inches (15.2 cm), and 8 inches (20.3 cm) over a period of 1 to 2 h. The distance between each cavaletti pole was the measured withers height of each dog. The dogs were allowed to rest for a minimum of 10 min between each increase in cavaletti pole height. The trial was considered valid if the dog had three feet on the walkway, did not pull on the leash, did not turn its head significantly off midline, and walked over the cavaletti poles one limb at a time. Each dog completed multiple trials until five valid trials were completed for each height.

Data were collected for dogs walking over the pressure-sensitive walkway (heretofore known as “walking gait”) and over sequentially increasing heights of cavaletti (2 inches, 4 inches, 6 inches, and 8 inches). The temporospatial data variables collected were gait velocity (gait distance [total distance walked on the PSW]/gait time [duration to walk the total distance on the PSW]), number of gait cycles per minute (frequency at which a dog completes its gait cycles within a minute), gait cycle duration (duration of one complete cycle of gait, which includes the time from the initial contact of one paw to the subsequent contact of the same paw), and gait time (duration to walk the total distance on the PSW). The ground reaction force variables collected for each limb were maximum peak pressure, stance time, maximum force, and vertical impulse. Maximum force and vertical impulse were measured as normalized values (% body weight (kg) and % body weight (kg) x seconds, respectively).

Statistical analysis was performed, and data were analyzed using mixed models general in NCSS 2019. TPS data were analyzed using a two-factor ANOVA. The normality of the errors was evaluated using histograms and normal probability plots and accepted. Sphericity (homogeneity of the variances of the differences) was addressed by assessing various repeated covariance patterns and selecting the best (first-order autogressive) using Akaike’s Information Criterion. Data were reported as mean +/− SD. The value of *p* < 0.05 was considered significant.

## 3 Results

In total, 32 dogs were evaluated. Of which, 25 dogs met the inclusion criteria. Seven dogs did not meet the inclusion criteria and were excluded from enrollment. Of the dogs excluded, one dog was determined to have neurologic dysfunction, three dogs had a withers height of <50 cm, and three dogs were not amenable to leash walking over the PSW. The study population included mixed breed (9), Australian Shepherd (3), Labrador Retriever (3), Doberman Pinscher (2), Golden Retriever (2), Border Collie (1), German Shepherd (1), Pitbull (1), Siberian Husky (1), Standard Poodle (1), and Visla (1). Three dogs were intact males, 14 dogs were neutered males, one dog was an intact female, and seven dogs were spayed females. The mean age of the dogs was 5.8 ± 2.9 years (range: 1.5–11 years). Mean weight and BCS were 27.5 ± 5.6 kg (range: 18–40.6 kg) and 5.28 ± 0.9 (range: 4.5–7.5), respectively. The mean withers height was 55.9 ± 4.8 cm (range: 50–65 cm).

### 3.1 Temporospatial variables

**Figure 2:**
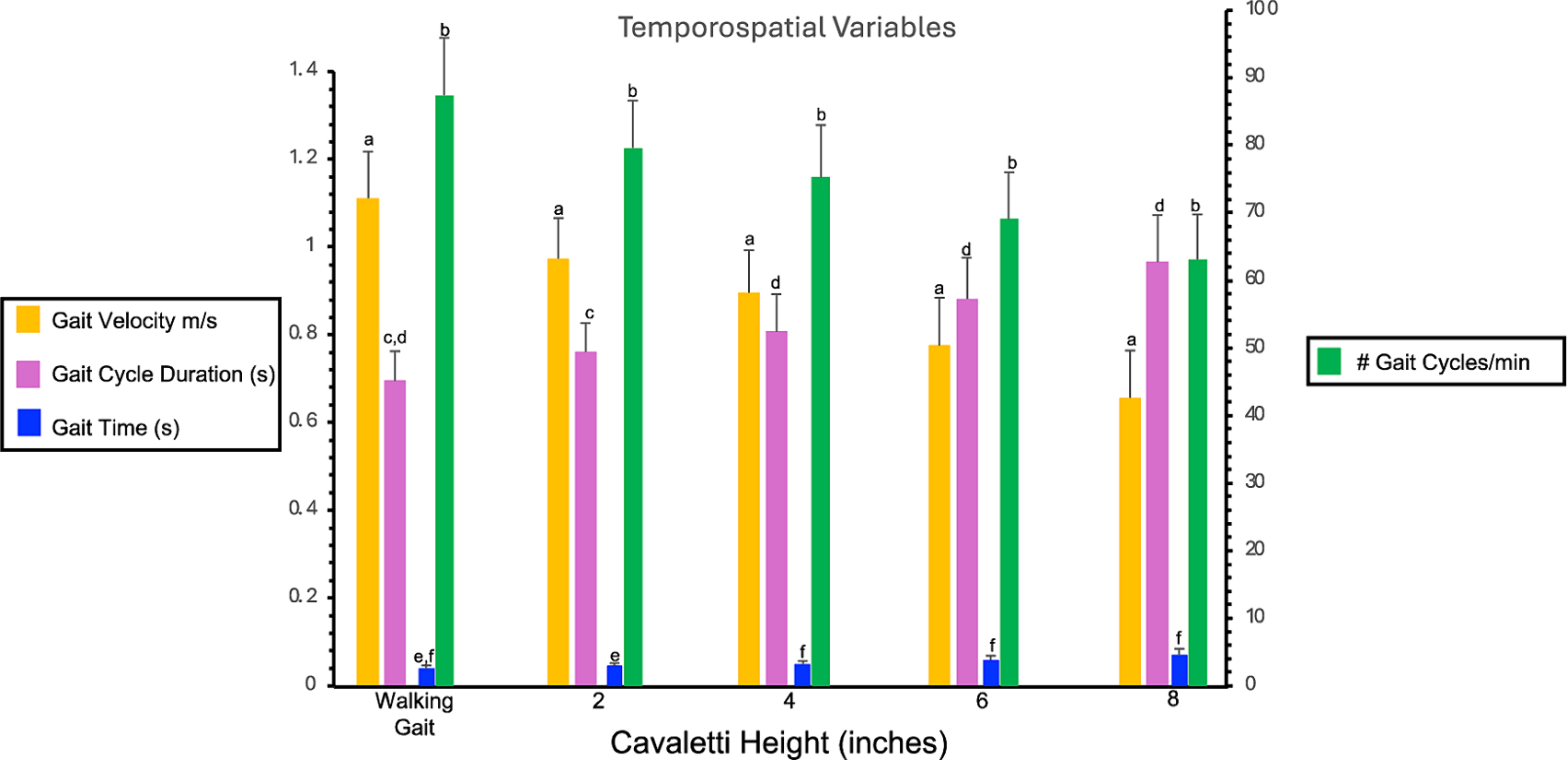
Bar graph illustrates the comparison of the effects of cavaletti pole height on gait velocity (m/s), number of gait cycles per minute, gait cycle duration (seconds), and gait time (seconds) as compared to a walking gait. Data represent mean ± SD. Gait velocity is gait distance (total distance walked on the PSW)/gait time (duration to walk the total distance on the PSW). Gait cycle duration is the duration of one complete cycle of gait, which includes the time from the initial contact of one paw to the subsequent contact of the same paw. Gait time is the duration to walk the total distance on the PSW. #gait cycles per minute is the frequency at which a dog completes its gait cycles within a minute.

Walking over cavaletti poles of increasing heights resulted in significant differences in gait velocity, number of gait cycles per minute, gait cycle duration, and gait time as compared to the same variables obtained for a walking gait. The gait velocity in dogs walking over 2″, 4″, 6″, and 8″ cavaletti heights was significantly decreased compared to a walking gait (*p* < 0 0.001, Table 1; Figure 2). The number of gait cycles per minute was also significantly decreased for all cavaletti heights compared to a walking gait (*p* < 0.001, Table 1; Figure 2). The converse was noted with both gait cycle duration and gait time. Increasing cavaletti height resulted in an increase in gait cycle duration for 2″ cavaletti height (*p* < 0.001) in addition to 4″, 6″, and 8″ heights (p < 0.001) compared to a walking gait (Table 1; Figure 2). Gait time was also significantly increased for 2″ (*p* = 0.004), 4″, 6″, and 8″ (p < 0.001) cavaletti height compared to a walking gait (Table 1; Figure 2).

**Table 1:** Comparison of the effects of cavaletti pole height on temporospatial measurements for dogs walked over a pressure sensitive walkway.

Variable	Walking Gait	2” cavaletti	4” cavaletti	6” cavaletti	8” cavaletti
Gait velocity (m/s)	1.11 ± 0.11^a^	0.97 ± 0.09^a^	0.89 ± 0.09^a^	0.77 ± 0.11^a^	0.66 ± 0.11^a^
No of gait cycles/min	87.40 ± 8.52^b^	79.65 ± 6.97^b^	75.26 ± 7.74^b^	69.05 ± 6.99^b^	63.09 ± 6.66^b^
Gait cycle duration (s)	0.69 ± 0.07^c^	0.76 ± 0.07^c^	0.81 ± 0.09^d^	0.88 ± 0.09^d^	0.97 ± 0.11^d^
Gait distance (m)	2.84 ± 0.09	2.84 ± 0.12	2.85 ± 0.09	2.76 ± 0.41	2.87 ± 0.09
Gait time (s)	2.54 ± 0.48^d^	2.97 ± 0.36^e^	3.24 ± 0.41^f^	3.78 ± 0.68^f^	4.55 ± 0.94^f^

Data represent mean ± SD. ^a,b^Significant difference for 2”, 4”, 6”, 8” cavaletti height compared to a walking gait (*p* < 0.001). ^c^Significant difference for 2” cavaletti height compared to a walking gait (*p* < 0.001). ^d^Significant difference for 4”, 6”, 8” cavaletti height compared to a walking gait (*p* < 0.001). ^e^Significant difference for 2” cavaletti height compared to a walking gait (*p* = 0.004). ^f^Significant difference for 4”, 6”, 8” cavaletti height compared to a walking gait (*p* < 0.001).

### 3.2 Ground reaction force variables

No observed differences were noted for maximum peak pressure (Table 2). Increases in stance times were observed for all limbs at every cavaletti height (Table 3). An increase in normalized maximum force was noted in the forelimbs but not in the hindlimbs (Table 4). Additionally, increases in normalized vertical impulse were observed in both the forelimbs and hindlimbs (Table 5). Due to the lack of consistent velocity across all test groups, no comparisons or inferences were made regarding these observations for ground reaction force variables.

**Table 2:** Observed maximum peak pressure (psi) for each limb.

Limb	Walking Gait	2”cavaletti	4”cavaletti	6”cavaletti	8”cavaletti
Left forelimb	45.17 ± 6.89	46.19 ± 6.98	47.78 ± 8.05	46.76 ± 7.23	47.16 ± 7.49
Right forelimb	47.44 ± 7.53^e^	49.06 ± 7.63	49.04 ± 7.55	50.08 ± 7.96	50.37 ± 7.19^e^
Left hindlimb	37.98 ± 6.79	38.66 ± 5.29	38.74 ± 5.19	38.56 ± 4.88	40.33 ± 5.78
Right hindlimb	40.61 ± 8.75	40.09 ± 5.59	40.33 ± 5.39	40.47 ± 5.13	40.99 ± 5.33

Data represent mean ± SD.

**Table 3:** Observed stance time (seconds) for each limb.

Limb	Walking Gait	2” cavaletti	4” cavaletti	6” cavaletti	8” cavaletti
Left forelimb	0.41 ± 0.05	0.45 ± 0.05	0.47 ± 0.06	0.52 ± 0.06	0.56 ± 0.08^j^
Right forelimb	0.41 ± 0.05	0.45 ± 0.05	0.48 ± 0.06	0.52 ± 0.07	0.57 ± 0.08^k^
Left hindlimb	0.39 ± 0.04	0.43 ± 0.04	0.46 ± 0.06	0.50 ± 0.06	0.54 ± 0.08^l^
Right hindlimb	0.40 ± 0.04	0.44 ± 0.05	0.47 ± 0.06	0.50 ± 0.06	0.54 ± 0.08^m^

Data represent mean ± SD.

**Table 4:** Observed normalized maximum force (%BW) for each limb.

Limb	Walking Gait	2” cavaletti	4” cavaletti	6” cavaletti	8” cavaletti
Left forelimb	56.27 ± 9.59	57.37 ± 8.62	60.01± 9.72	60.63 ± 9.72	60.79 ± 9.08
Right forelimb	58.90 ± 9.72	60.53 ± 8.56	61.80 ± 9.18	64.11 ± 10.78	63.75 ± 9.85
Left hindlimb	44.22 ± 7.79	44.24 ± 8.40	44.59± 8.73	44.24 ± 8.23	45.14 ± 7.78
Right hindlimb	45.18 ± 8.44	44.80 ± 7.88	45.17 ± 7.70	45.71 ± 7.99	47.40 ± 8.56

Data represent mean ± SD.

**Table 5:** Observed normalized vertical impulse (%BW x sec) for each limb.

Limb	Walking Gait	2” cavaletti	4” cavaletti	6” cavaletti	8” cavaletti
Left forelimb	16.32 ± 3.43	18.43 ± 3.37^l^	20.35 ± 4.30	22.17 ± 3.33	23.94 ± 4.03
Right forelimb	17.26 ± 3.03	19.49 ± 3.12	20.93 ± 3.93	23.50 ± 3.87	25.67 ± 4.40
Left hindlimb	11.88 ± 1.9	12.92 ± 2.11	13.73± 2.30	14.58 ± 2.05	16.14 ± 2.81
Right hindlimb	12.59 ± 2.28	13.42 ± 2.23	14.15 ± 2.18	15.22 ± 1.98	16.87 ± 2.72

Data represent mean ± SD.

## 4 Discussion

Cavaletti poles are a common component of veterinary rehabilitation programs. These are utilized to strengthen the muscles, promote weight bearing, improve balance and proprioception, and increase the joint active range of motion. This investigational study aimed to assess the impact of walking over cavaletti poles of varying heights on temporospatial and ground reaction variables in healthy subjects, with the ultimate goal of enhancing comprehension regarding their potential utility in comparative rehabilitative therapy programs for patients with orthopedic and neuromuscular challenges.

The results of the current study demonstrated that increasing cavaletti pole height has an effect on temporospatial variables. The true effect on ground reaction force variables cannot be determined due to the lack of consistent velocity across all test groups. We therefore partially accept and partially reject our hypothesis.

Both gait velocity and the number of gait cycles per minute decreased significantly for all cavaletti pole heights when compared to a walking gait. The converse was noted for gait cycle duration and gait time, in that both variables increased. Each incremental increase in cavaletti height resulted in a corresponding decrease in gait velocity and the number of gait cycles and an increase in gait cycle duration and gait time. In human and animal studies for which obstacle walking has been investigated, both decreased velocity and cadence have been reported. Dogs walking over two 13-cm (5.1 inches) height obstacles, separated by 35 cm (13.8 inches), resulted in a significantly slower center of pressure (COP) speed as compared to a walking gait (62). A human study yielded comparable results, showing reduced obstacle-crossing speed corresponding to increased obstacle height (42). In the present study, negotiating multiple sequential obstacles resulted in changes to temporospatial variables presumably required to enable the successful navigation of the obstacles.

Gait velocity has been shown to influence ground reaction forces (63–66). Therefore, the use of a constant velocity has been recommended to minimize data variability (15). Studies have documented that as gait velocity increases, peak vertical forces increase and stance time decreases. A gait velocity ranging between 0.8 and 1.3 m/s has been reported for walking (15, 18). The dogs in this study were allowed to walk over the cavaletti poles at a comfortable pace, mirroring the approach typically adopted in clinical practice during therapeutic exercise. When using cavaletti poles in a therapeutic exercise program, dogs are typically walked slowly to encourage weight bearing on all limbs. A faster pace can often lead to the dog hopping or jumping over obstacles, avoiding the need to place the affected limb on the ground, which negates the purpose of the exercise. Therefore, to replicate clinical practice, we did not force the dogs to walk faster. Allowing each dog to navigate the obstacles at their own pace resulted in a decrease in the walking gait velocity with each incremental increase in cavaletti height. Ideally, the dogs would have walked at a set velocity for each cavaletti height. However, to maintain a constant velocity for each cavaletti height, the dogs would have needed to be led at a faster pace. Based on human and rodent studies investigating obstacle walking, a decrease in velocity was anticipated. However, the magnitude of this decrease and the specific velocity range for canine ambulation over multiple obstacles set at specific heights were unknown. The inability of the dogs to maintain a consistent velocity across all cavaletti heights highlighted the impact of increasing cavaletti height. The slower velocities observed with increasing cavaletti height suggest modifications to walking gait patterns to successfully navigate the obstacles. Gait velocity is a critical variable in canine gait analysis as it directly affects ground reaction force variables. Consequently, in this study, the increasing cavaletti height directly affected the velocity. The velocity ranges acquired for each cavaletti height may serve as a foundation for further investigation of our understanding of the dynamics of the canine gait when walking over obstacles. This information may be beneficial for therapeutic and rehabilitation purposes as controlling gait velocity may help manage the forces exerted on the dog’s limbs, which is important for dogs recovering from injuries or surgeries. This information will also be valuable for future studies related to velocity and TPS and GRF variables.

Conclusions regarding the direct effect of cavaletti height on ground reaction forces cannot be drawn from the data obtained in this study. Maximum peak pressure, stance time, normalized maximum force, and normalized vertical impulse are all affected by and correlated with velocity. It is unclear whether the increases in stance time, normalized maximum force, and normalized vertical impulse observed in this study are due to the decrease in velocity, the cavaletti height, or a combination of both. To better elucidate the effects of cavaletti pole height on ground reaction forces, maintaining the same walking velocity for all cavaletti heights would be necessary.

The current study presents several limitations. Most notably, the lack of consistent velocity for all cavaletti height trials introduced variability. The absence of established velocity ranges for dogs walking over each cavaletti height prevented the assignment of a specific velocity for a particular cavaletti height. These differing velocities serve as a confounding variable when interpreting the GRF data. A comparison to a walking gait within the velocity range corresponding to a specific cavaletti height would further clarify the effects of the cavaletti height on TPS and GRF variables.

A heterogeneous population of medium to large dogs was used, resulting in a 15-cm range in withers height. A more clinically homogeneous study population might have led to reduced variability in the outcome variables. Although dogs underwent assessment for overt orthopedic disease, subclinical orthopedic diseases, such as osteoarthritis, cannot be entirely ruled out.

Radiographs could have been obtained for a more comprehensive evaluation of forelimb and hindlimb joints to exclude dogs with orthopedic disease. However, radiographic disease evidence may or may not correlate with clinical disease or soundness (67). Olsson et al. reported that clinical signs are often unrelated to radiographic severity (68). This disparity has been explored through force-plate analysis, which highlighted a poor correlation between radiographic osteoarthritis (OA) and limb function (69, 70), as well as clinician- and owner-reported pain severity, which again were not associated with radiographic severity (71). Furthermore, in comparison to human medicine, no single clinical scoring system has been accepted as the standard of care in the diagnosis of canine OA with radiography (72, 73).

Additionally, the dogs were consistently led from their right side (handler’s left side) and always in the same direction over the pressure-sensitive walkway (PSW). Alternating the side from which the dogs were led and the walking direction may have provided additional insights. The sequential increase in cavaletti height was not randomized. Randomizing the height order could have mitigated potential biases. Finally, all trials were conducted within a single day, possibly impacting fatigue levels and performance consistency.

The primary aim of this study was to gain a more global understanding of both TPS and GRF in healthy dogs navigating multiple obstacles. Therefore, an in-depth examination of the dynamics of the leading and trailing forelimbs and hindlimbs was not performed. Consequently, a limitation of this study arises from the absence of detailed information regarding the leading and trailing limbs of dogs while navigating vertical obstacles. For a comprehensive understanding of the kinetics involved in walking over multiple obstacles, further research is warranted to elucidate the distinct effects on both the leading and trailing forelimbs and hindlimbs.

This investigation was conducted in a cohort of healthy dogs without overt signs of orthopedic disease or neurologic dysfunction, walking on a flat surface in a straight line. Further research is required to delve deeper into the temporospatial (TPS) and ground reaction force (GRF) variables during obstacle walking compared to walking at a slower velocity within the range corresponding to the obstacle height. Subsequent studies could also explore the influence of cavaletti height in patients with pathological conditions and varied orientations in healthy individuals as they transition back to sporting activities.

Despite the limitations of this study, the data do provide initial insight regarding walking exercises over cavaletti poles. Increasing heights resulted in slower walking velocities. This information is applicable and relevant in the clinical setting. To facilitate weight bearing and ensure the exercise is performed correctly, the height of the cavaletti poles can be increased, encouraging the dog to walk and step over each obstacle and preventing the dog from moving at a faster pace. Additional studies are warranted to further investigate the relationship between cavaletti height and ground reaction forces.

## Data availability statement

The raw data supporting the conclusions of this article will be made available by the authors, without undue reservation.

## Ethics statement

The animal studies were approved by Oklahoma State University Animal Care and Use Committee. The studies were conducted in accordance with the local legislation and institutional requirements. Written informed consent was obtained from the owners for the participation of their animals in this study.

## Author contributions

CB: Conceptualization, Data curation, Formal analysis, Funding acquisition, Investigation, Methodology, Project administration, Resources, Supervision, Visualization, Writing – original draft, Writing – review & editing. AL: Writing – review & editing, Conceptualization, Methodology. TM: Data curation, Writing – review & editing.

## Footnotes

^1^Tough1 Miniature Sure Measure Height Standard, JT International, Indianapolis, IN.

^2^5-Tile High Resolution Strideway System, Tekscan Inc., South Boston, MA.

^3^Canine Pro-Cones, Balanced Canine Products, Denver, CO.
